# Prognostic significance of chest CT severity score in mortality prediction of COVID-19 patients, a machine learning study

**DOI:** 10.1186/s43055-023-01022-z

**Published:** 2023-04-20

**Authors:** Seyed Salman Zakariaee, Aza Ismail Abdi, Negar Naderi, Mashallah Babashahi

**Affiliations:** 1 Department of Medical Physics, Faculty of Paramedical Sciences, Ilam University of Medical Sciences, Ilam, Iran; 2 Department of Radiology, Erbil Medical Technical Institute, Erbil Polytechnic University, Erbil, Iraq; 3 Department of Midwifery, Faculty of Nursing and Midwifery, Ilam University of Medical Sciences, Ilam, Iran; 4 Department of Pathology, Faculty of Paramedical Sciences, Ilam University of Medical Sciences, Ilam, Iran

**Keywords:** Chest CT severity score, COVID-19, CT-SS, Machine learning, Mortality prediction

## Abstract

**Background:**

The high mortality rate of COVID-19 makes it necessary to seek early identification of high-risk patients with poor prognoses. Although the association between CT-SS and mortality of COVID-19 patients was reported, its prognosis significance in combination with other prognostic parameters was not evaluated yet.

**Methods:**

This retrospective single-center study reviewed a total of 6854 suspected patients referred to Imam Khomeini hospital, Ilam city, west of Iran, from February 9, 2020 to December 20, 2020. The prognostic performances of k-Nearest Neighbors (kNN), Multilayer Perceptron (MLP), Support Vector Machine (SVM), and J48 decision tree algorithms were evaluated based on the most important and relevant predictors. The metrics derived from the confusion matrix were used to determine the performance of the ML models.

**Results:**

After applying exclusion criteria, 815 hospitalized cases were entered into the study. Of these, 447(54.85%) were male and the mean (± SD) age of participants was 57.22(± 16.76) years. The results showed that the performances of the ML algorithms were improved when they are fed by the dataset with CT-SS data. The kNN model with an accuracy of 94.1%, sensitivity of 100. 0%, precision of 89.5%, specificity of 88.3%, and AUC around 97.2% had the best performance among the other three ML techniques.

**Conclusions:**

The integration of CT-SS data with demographics, risk factors, clinical manifestations, and laboratory parameters improved the prognostic performances of the ML algorithms. An ML model with a comprehensive collection of predictors could identify high-risk patients more efficiently and lead to the optimal use of hospital resources.

## Background

Coronavirus disease 2019 (COVID-19) is a life-threatening infection caused by severe acute respiratory syndrome coronavirus 2 (SARS-CoV-2) [1]. Despite all the preventive and lockdown measures taken by governments, the COVID-19 outbreak continues to spread aggressively worldwide and an exponential daily increasing number of infected cases is reported. The complex and highly contagious nature of COVID-19 has made this infection a serious global health concern and a notable pandemic [2–5]. The clinical outcomes of COVID-19 range from asymptomatic, mild or moderate symptoms to serious complications and death in some cases [6].

This virus has made a tremendous impact on the health status of people all over the world and caused a significant number of deaths. Approximately 20% of COVID-19 patients need to be hospitalized [7] and the pooled case fatality rate (CFR) of these patients is 13%. While, CFR in patients admitted in intensive care unit (ICU) would be 37.0% [8]. The high mortality rate of COVID-19 particularly for elderly populations and patients with underlying comorbidities including cardiopulmonary diseases, cancer, hypertension, diabetes, and low immune functions makes it necessary to seek early identification of high-risk patients with poor prognoses. Due to the unpredictability of the disease behavior and courses, the prognosis of disease progression in hospitalized patients and the identification of patients prone to rapid deterioration is a challenging clinical problem. Clinicians and health policymakers have commonly used predictions made by different statistical models to deal with these challenges [9, 10]. As a good alternative, artificial intelligence (AI) may be a helpful tool to identify patients with high-risk of mortality. AI is a noninvasive digital technology that can facilitate accurate and timely identification of high-risk patients. Machine learning (ML) is a subset of AI that looks for hidden and previously unknown patterns from large sets of data [11]. In the prior studies, ML-based models were evaluated to predict the risk of patient deterioration and death. These studies mainly used demographics, risk factors, clinical manifestations, and laboratory results [12–15]. In recent meta-analysis studies, it was shown that chest computed tomography severity score (CT-SS) is an appropriate prognostic factor for mortality prediction in COVID-19 patients [16, 17]. Therefore, it might improve the prognostic performances of the ML algorithms for predicting clinical outcomes of patients with COVID-19 pneumonia. In this study, we evaluated the prognostic significance of CT-SS in the mortality prediction of COVID-19 patients using the selected well-known ML algorithms. The prognostic performances of four ML algorithms including kNN, MLP, SVM, and J48 decision tree algorithms are assessed in the presence and absence of CT-SS data.

## Methods

### Dataset description

**Fig. 1:**
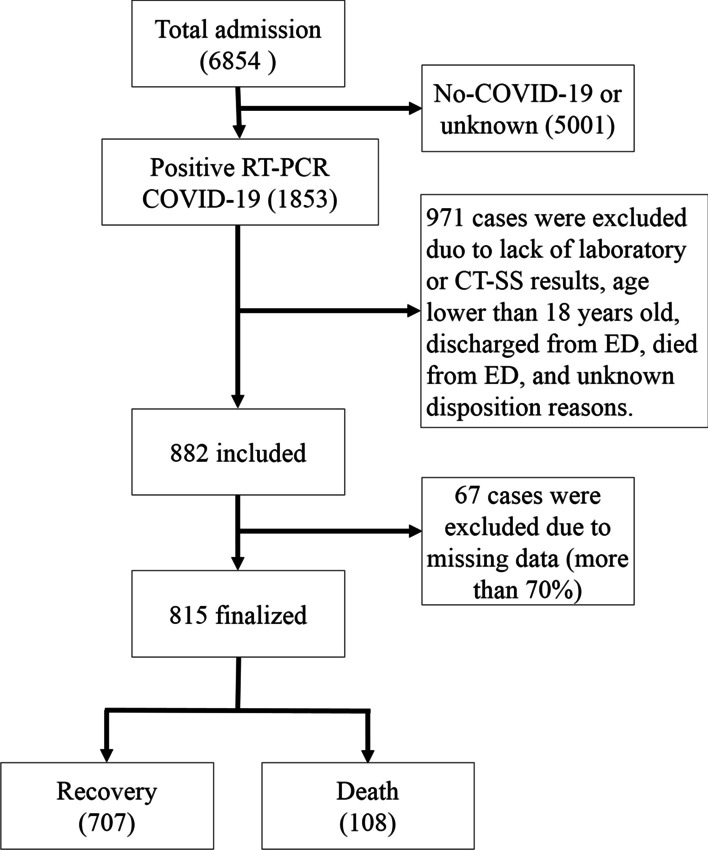
Flowchart describing patient selection

This retrospective single-center study was conducted in 2022 to predict mortality in COVID-19 patients based on four popular ML algorithms. This study reviewed a hospital-based COVID-19 registry database from Imam Khomeini hospital, Ilam city, west of Iran, from February 9, 2020 to December 20, 2020. During this period, a total of 6854 suspected cases were referred to Imam Khomeini Hospital’s ambulatory and emergency departments (EDs), of whom 1853 cases were introduced as positive RT-PCR COVID-19, 2472 as negative, and 2529 as unknown. Only the patients with positive RT-PCR tests were included in the study (Fig. 1).

The included cases were linked to 55 primary features in six main classes including patient’s demographics (five features), clinical pictures (14 features), history of personal diseases/comorbidity (seven features), laboratory results (26 features), CT-SS (one feature), and an output variable (0: survived and 1: deceased) (see Table 1).

**Table 1:** The most important variable at *P* < 0.01 using the Chi-squared test

No	Features name	Variable type	Frequency or mean ± SD	*Χ*^2^	*P *value	No	Features name	Variable type	Frequency or mean ± SD	*Χ*^2^	*P *value
1	Age	Numeric	57.22 ± 16.76	29.520	< 0.001	15	Absolute lymphocyte count	Numeric	2.21 ± 1.17	56.562	< 0.001
2	Sex	Nominal	Female(368) Male(447)	9.397	0.002	16	Absolute neutrophil count	Numeric	7.37 ± 1.73	10.290	0.006
3	Cough	Nominal	Haven’t (209) Have (606)	23.077	< 0.001	17	Calcium	Numeric	9.52 ± 0.80	14.471	0.001
4	Underlying disease	Nominal	Haven’t (328) Have (487)	20.450	< 0.001	18	Phosphor	Numeric	3.48 ± 0.48	11.612	0.003
5	Heart disease	Nominal	Haven’t (648) Have (167)	20.921	< 0.001	19	Blood urea nitrogen	Numeric	39.69 ± 25.56	18.258	< 0.001
6	Hypertension	Nominal	Haven’t (571) Have (244)	8.164	0.004	20	Total bilirubin	Numeric	0.62 ± 0.50	21.537	< 0.001
7	Diabetes	Nominal	Haven’t (634) Have (181)	7.496	0.006	21	Serum albumin	Numeric	4.09 ± 0.46	15.818	< 0.001
8	Neurological disease	Nominal	Haven’t (776) Have (39)	7.968	0.005	22	Glucose	Numeric	137.45 ± 83.50	15.005	0.001
9	Cancer	Nominal	Haven’t (791) Have (24)	17.369	< 0.001	23	Creatinine kinase	Numeric	137.68 ± 234.68	27.031	< 0.001
10	Serum creatinine	Numeric	1.21 ± 0.56	55.795	< 0.001	24	Activated partial thromboplastic time	Numeric	28.26 ± 12.54	17.172	< 0.001
11	RBC	Numeric	4.58 ± 0.74	18.665	< 0.001	25	Prothrombin time	Numeric	12.76 ± 2.38	30.123	< 0.001
12	WBC	Numeric	7719.02 ± 3939.55	72.375	< 0.001	26	Hypersensitive troponin	Nominal	Normal (791) Abnormal (24)	22.836	< 0.001
13	Hematocrit	Numeric	39.61 ± 6.67	19.847	< 0.001	27	CTSS	Numeric	11.18 ± 5.28	71.482	< 0.001
14	Hemoglobin	Numeric	13.44 ± 2.15	23.882	< 0.001						

The severity of the pulmonary involvement for each patient was evaluated using the chest CT score. Five lung lobes were visually scored as 0 (no involvement), 1 (less than 5% involvement), 2 (5%–25% involvement), 3 (26%–49% involvement), 4 (50%– 75% involvement), and 5 (50%– 75% involvement). The sum of these scores yielded the total CT-SS ranging from 0 to 25. Two radiologists separately reviewed all CT images. Any disagreements were resolved through consulting with an attending radiologist with 23 years of experience.

### Data pre-processing

In the data mining process, the use of raw data reduces the efficiencies of the algorithms and the achieved results would have poor qualities. The refined information extracted from the raw data considerably improves the model’s ability to learn. Therefore, the data pre-processing would be a crucial step before the training of the model. This approach resolves inconsistencies and addresses irrelevant, redundant, and unreliable data [18].

In this study, the incomplete records with many missing values (more than 70%) were excluded from the dataset. Noisy and abnormal values, errors, and meaningless data were checked by two authors (MB and SSZ). The missing cells of continuous and discrete variables were imputed by mean and mode values, respectively.

The schematic of the study inclusion and exclusion criteria is shown in Fig. 1. The final sample size was 815 COVID-19 patients. The refined dataset is significantly imbalanced in terms of the number of records in outcome classes. It contains 707 and 108 cases in the alive and death classes, respectively. This problem would cause delivering results biased toward the dominant class. In this study, the synthetic minority over-sampling technique (SMOTE) method was used to deal with the unbalanced dataset (https://imbalanced-learn.org/stable/).

### Feature selection

The feature selection process is a beneficial statistical method commonly used in forecasting, pattern recognition, and classification modeling for determining the most important variables highly correlated with the target variable [19].

This statistical approach prevents overfitting of the data mining algorithms, results in better classification of the data and evaluation of fewer numbers of variables for work simplification [20]. In this study, the independence test of Chi-square was used for weighting the features based on their importance in the mortality prediction of COVID-19 patients. The Chi-square coefficient was calculated by the following equation:

1 $$x^{2} = \frac{{\left( {O_{i} - E_{i} } \right)^{2} }}{{E_{i} }}$$

where *O*_*i*_ and *E*_*i*_ are the observed and expected variables, respectively. The SPSS software (version 23) was used to determine the importance of the variables for mortality prediction of COVID-19 patients. In this study, *P* < 0.01 was regarded as the significant level.

### Model development

In this study, four well-known ML classification methods including kNN, MLP, SVM, and J48 decision tree algorithms were used to predict the mortality of the patient with confirmed COVID-19. These algorithms were implemented using Waikato Environment for Knowledge Analysis (Weka) software (version 3.9.2, University of Waikato, New Zealand). In the performance evaluation of the developed classifiers, tenfold cross-validation method was used. In this approach, the data set would be divided into ten subsets and all models run ten times. Each time, one subset was considered as test data and the remaining nine subsets would be training datasets. The results of these ten evaluations are mixed to render the performance metrics. Therefore, tenfold cross-validation method would be a preferred technique due to its relatively low-level bias and variation. The performances of these classification algorithms were determined in terms of accuracy, precision, sensitivity, specificity, F-measure, and area under the ROC curve (AUC).

ML algorithms were applied to datasets with and without CT-SS data to determine the prognostic significance of CT-SS in the mortality prediction of COVID-19 patients.

### Ethical considerations

This study was approved by the medical ethical committee (approved number: IR.MEDILAM.REC.1401.255). The unique identifying information of the patients was concealed to protect the privacy and confidentiality of the patients.

## Results

A total of 6854 suspected patients was registered in the Ilam CoV registry database. After applying exclusion criteria including negative RT-PCR COVID-19 test, age lower than 18 years old, discharged or death from the emergency department, missing data more than 70%, noisy and abnormal values, unknown dispositions, and lack of laboratory or CT-SS data, 815 hospitalized cases were entered into the study. Of these, 447(54.85%) were male and the mean (± SD) age of participants was 57.22(± 16.76) years. Out of 815 included patients, 707 patients recovered and 108 (13.3%) deceased. The number of records in the deceased class was raised to 707 after balancing the dataset.

### Feature selection

After utilizing the independence test of Chi-square to determine the importance of the variables for the mortality prediction of COVID-19 patients, 27 predictors were chosen as the most important and relevant features. The list of the most important variables and results of the independence test of Chi-square are demonstrated in Table 1. These features were used as the inputs for all ML algorithms. These features included demographics, risk factors, clinical manifestations, laboratory tests, and imaging results.

### Evaluation of the developed models

In this study, the predictive models were built using four well-known ML algorithms including kNN, MLP, SVM, and J48 decision tree. The subsets of features selected using the independence test of Chi-square were used to develop COVID-19 mortality prediction models. The ML algorithms were separately trained using the datasets with and without CT-SS data. The performances of these models were evaluated using sensitivity, specificity, accuracy, precision, F-measure, and AUC metrics. The results of the performance evaluation for the developed models using the datasets with and without CT-SS data are listed in Table 2. The results showed that the kNN algorithm yielded better performance to predict the mortality of COVID-19 patients than other ML algorithms. Sensitivity, specificity, accuracy, precision, F-Measure, and AUC of the kNN algorithm fed by the dataset without CT-SS data were 100.0%, 87.0%, 93.5%, 88.5%, 93.9%, and 97.5%, respectively. For the dataset with CT-SS data, the kNN algorithm reached 100.0% sensitivity, 88.3% specificity, 94.1% accuracy, 89.5% precision, 94.5% F-Measure, and an AUC of 97.2%.

**Table 2:** Performances of ML algorithms fed by the datasets with and without CT-SS data

ML algorithm	Sensitivity		Specificity		Accuracy		Precision		F-Measure		AUC	
	Without CT-SS	With CT-SS	Without CT-SS	With CT-SS	Without CT-SS	With CT-SS	Without CT-SS	With CT-SS	Without CT-SS	With CT-SS	Without CT-SS	With CT-SS
Decision tree	0.979	0.984	0.844	0.849	0.912	0.917	0.863	0.867	0.917	0.922	0.931	0.939
SVM	0.808	0.830	0.765	0.793	0.786	0.812	0.775	0.801	0.791	0.815	0.786	0.812
MLP	0.979	0.984	0.895	0.911	0.937	0.948	0.903	0.917	0.940	0.950	0.962	0.970
kNN	1.000	1.000	0.870	0.883	0.935	0.941	0.885	0.895	0.939	0.945	0.975	0.972

**Fig. 2:**
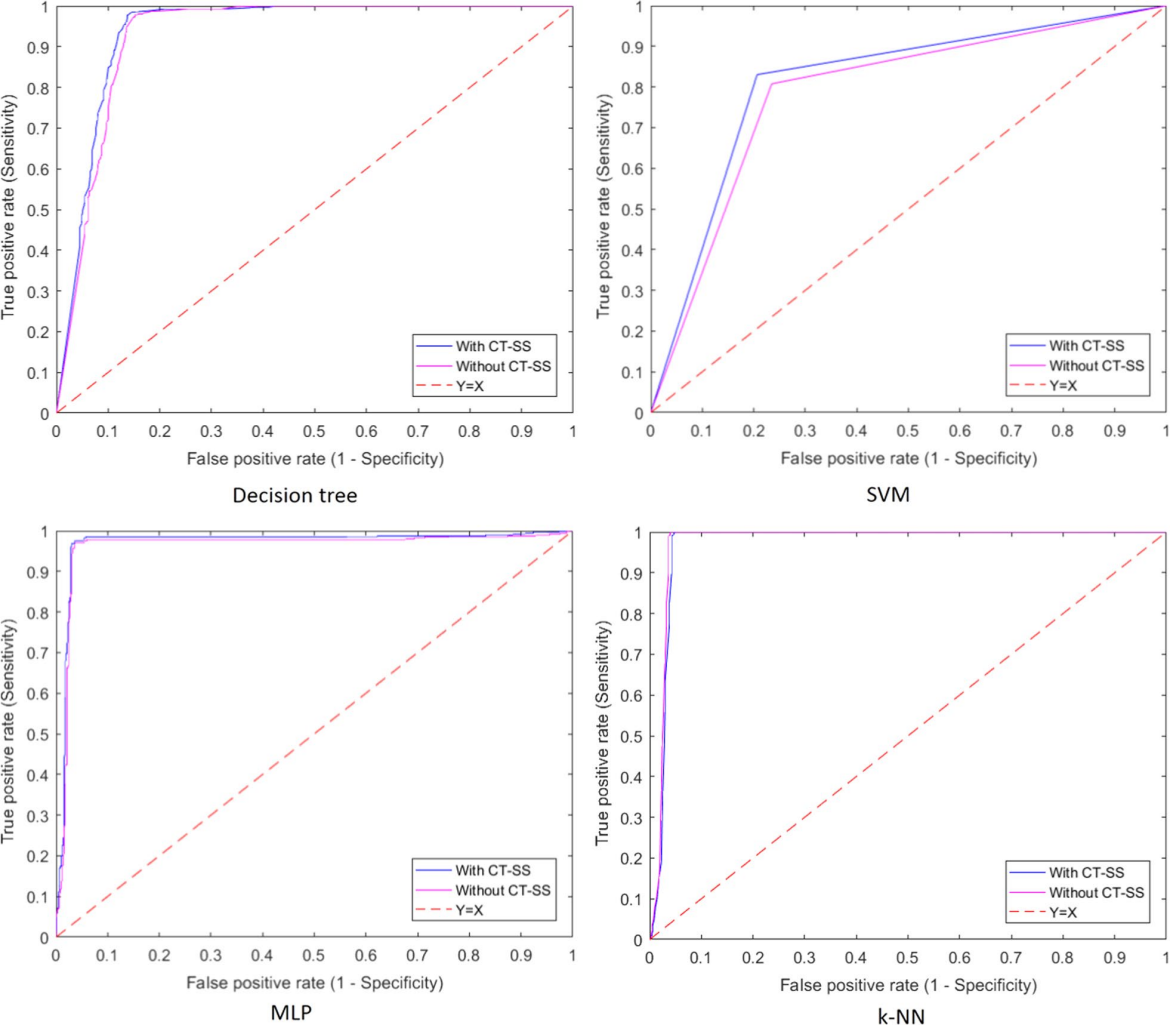
ROC curves for ML algorithms fed by the datasets with and without CT-SS data

Figure 2 depicts the ROC curves for the selected ML algorithms fed by the datasets with and without CT-SS data, separately.

The results showed that the performances of the algorithms were improved when they are fed by the dataset with CT-SS data.

## Discussion

During the major outbreak of COVID-19, a timely and accurate prognosis of disease progression and clinical outcomes of patients could provide better guidelines for the management of the disease. An efficient predictive model to identify high-risk patients could have a crucial role in risk stratification for the allocation of finite resources and improved patient survival probability. For appropriate preparedness against this global pandemic, new technologies and AI-based solutions have been suggested for diagnostic, screening, monitoring, and prognostic goals. In these studies, various models were evaluated and the most relevant predictive parameters were reported [21–27].

CT-SS is a promising prognostic factor for mortality prediction in COVID-19 patients that evaluate the severity and extent of pulmonary involvement. A recent meta-analysis study showed that CT-SS index with and without an optimal cutoff was positively associated with mortality of COVID-19 patients (OR 7.124; 95% CI 5.307–9.563 and OR 1.244; 95% CI 1.157–1.337, respectively) [17]. Sensitivity, specificity, and AUC of this predictive parameter were 0.67 (95%CI: 0.59–0.75), 0.79 (95%CI: 0.74–0.84), and 0.8248, respectively [16]. Although the association between CT-SS and mortality of COVID-19 patients was reported [16, 17], its prognosis significance in combination with other prognostic parameters was not evaluated yet. Therefore, in this study, kNN, MLP, SVM, and J48 decision tree algorithms were developed based on the most relevant features in determining the risk of COVID-19 mortality. The prognostic importance of the parameters in the mortality prediction of COVID-19 patients was determined using the independence test of Chi-square. Some features such as age, cough, underlying disease, serum creatinine, and CT-SS were of the highest importance. Relevant predictors of deterioration and mortality risks of COVID-19 patients were also reported by several studies [9, 10, 12, 13, 28–39]. The most relevant predictive features reported by these studies are similar to our findings. On the other hand, some features such as smoking, alcohol consumption, drug addiction, platelet count, and alanine aminotransferase (ALT) had a low weight of significance in predicting COVID-19 mortality. It should be noted that these variables with the lowest significance in predicting COVID-19 mortality are consistent with those reported by the study of Moulaei et al. [15]. Although these factors have a considerable clinical role in the success of treatment, many of these features can be ignored in the ML approach and poor outcomes of the infected patients could be efficiently predicted with fewer factors.

In the next step, the prognostic performances of the selected ML models were evaluated based on a dataset of laboratory-confirmed COVID-19 hospitalized patients with and without CT-SS data. The results showed that the integration of CT-SS data with demographics, risk factors, clinical manifestations, and laboratory parameters improved the prognostic performances of the ML models. Also, the kNN model had the best performance among the other three ML techniques with the accuracy of 94.1%, sensitivity of 100.0%, precision of 89.5%, specificity of 88.3%, and AUC around 97.2%. MLP and J48 decision tree models had a good prediction performance (AUCs > 93%), and their prognostic efficiency was better than the SVM model.

In several studies, ML techniques were evaluated in predicting mortality of the COVID-19 patients. In Moulaei et al. study [14], J48 decision tree, MLP, kNN, random forest (RF), and SVM data mining models were evaluated to predict the mortality of Covid-19 patients. Sixteen factors including demographics, risk factors, and clinical manifestations were used to predict the mortality of COVID-19 patients. The retrospective analysis of the data of 850 COVID-19 hospitalized patients showed that all ML algorithms have an acceptable prognostic performance (AUCs > 96%). RF model showed a slightly better performance and SVM was the weakest method in predicting mortality. In a similar paper conducted by Moulaei K et al. [15], mortality prediction of seven ML algorithms including the J48 decision tree, RF, kNN, MLP, Naïve Bayes (NB), eXtreme gradient boosting (XGBoost), and logistic regression (LR) models were evaluated. The results of this study had also shown that the RF model with the accuracy of 95.03%, sensitivity of 90.70%, precision of 94.23%, specificity of 95.10%, and AUC of 99.02 had better performance than other ML algorithms. In both studies, after the random forest model, kNN, MLP, and J48 decision tree algorithms had, respectively, the best prognostic performances. Their results were in close agreement with our findings.

In another study, the performances of LR, RF, SVM, and XGBoost algorithms for mortality prediction of COVID-19 patients were evaluated by Yadaw et al. [13]. In this study, data of 3841 confirmed COVID-19 patients (demographics, risk factors, and clinical manifestations) were analyzed and the results showed that the XGBoost algorithm with AUC of 91% was the best predictive model among all the models. In Gao et al. study [12], the retrospective analysis of 2520 COVID-19 hospitalized patients demonstrated that the model developed by the neural network (NN) had better performance in predicting COVID-19 patient’s physiological deterioration and death than LR, SVM, and gradient boosted decision tree(AUC = 97.60%).

These studies showed that the ML approach can help healthcare providers, clinicians, and health policymakers to timely predict the deterioration of the patient's condition and reduce the severe complications and the resulting mortalities. The integration of CT-SS data with demographics, risk factors, clinical manifestations, and laboratory parameters would improve the performances of the ML models for mortality prediction of COVID-19 patients and increase the survival rate of the patients. An ML model with this comprehensive collection of predictors could identify high-risk patients more efficiently and lead to the optimal use of hospital resources.

## Limitations

This study had several limitations that must be addressed. (1) There were irregularities and imbalances in the registered data. Thus, noise and inadequate records were eliminated as much as possible. (2) As was mentioned, there were significantly higher numbers of records in the survived group than in the dead class (108 vs. 707). To solve this problem, SMOTE was used to minimize the bias via class balancing. (3) Unlike the prior studies in which there is a lack of radiological and imaging indicators; in this study, CT-SS data were used along with the demographics, risk factors, clinical manifestations, and laboratory results. Therefore, a more comprehensive collection of features was achieved that could enhance the prognostic performances of the models. (4) This is a retrospective single-center study that was conducted using the database collected at a designated referral hospital to deliver special healthcare services for COVID- 19 patients. Further studies need to be carried out with bigger and multicenter databases to perform the external validation of the proposed model. (5) In this study, clinical variables available at the initial time of admission were used to predict the mortality of COVID-19 patients. The time span from infection to admission that would affect the features was unclear. The evaluation of dynamic variations in the features could help for more efficient identification of patients with poor outcomes. 6) The prognostic performances of four well-known ML algorithms were evaluated. In the future, the performance accuracy of algorithms other than these algorithms would be compared to determine the best model to predict the mortality of COVID-19 patients.

## Conclusions

In this study, we evaluated the prognostic significance of CT-SS in the mortality prediction of COVID-19 patients using four well-known ML algorithms including kNN, MLP, SVM, and J48 decision tree algorithms in the presence and absence of CT-SS data. The results showed that the performances of the algorithms were improved when they are fed by the dataset with CT-SS data and the kNN algorithm yielded better performance to predict the mortality of COVID-19 patients than other ML algorithms. An ML model with a comprehensive collection of predictors could identify high-risk patients more efficiently and lead to the optimal use of hospital resources. This optimal predictive approach can help healthcare providers, clinicians, and health policymakers to timely predict the deterioration of the patient's condition and reduce the severe complications and the resulting mortalities.

## Data Availability

All data generated and analyzed during the current study are not publicly available but are available from the corresponding author on reasonable request and Ilam University of Medical Sciences’ approval.

## Abbreviations

COVID-19: Coronavirus disease 2019
SARS-CoV-2: Severe acute respiratory syndrome coronavirus 2
CT-SS: Chest CT severity score
CT: Computed Tomography
RT-PCR: Reverse-transcriptase polymerase chain reaction
ROC: Receiver operator characteristic curve
AUC: Area under the ROC curve
ML: Machine learning
k-NN: K-nearest neighborhood
MLP: Multilayer Perceptron
WEKA: Waikato Environment for Knowledge Analysis
SMOTE: Synthetic minority over-sampling technique.
OR: Odds ratio
